# Retrospective record review in proactive patient safety work – identification of no-harm incidents

**DOI:** 10.1186/1472-6963-13-282

**Published:** 2013-07-22

**Authors:** Kristina Schildmeijer, Maria Unbeck, Olav Muren, Joep Perk, Karin Pukk Härenstam, Lena Nilsson

**Affiliations:** 1 School of Health and Caring Sciences, Faculty of Health and Life Sciences, Linnaeus University, Kalmar, Sweden; 2 Department of Clinical Sciences, Karolinska Institutet, Danderyd Hospital, Division of Orthopaedics, Stockholm, Sweden; 3 Karolinska Institutet, Medical Management Centre, Stockholm, Sweden; 4 Division of Paediatrics, Astrid Lindgren Children’s Hospital, Karolinska University Hospital, Stockholm, Sweden; 5 Department of Anesthesia and Intensive Care, County Council of Östergötland, Linköping University, Linköping, Sweden

**Keywords:** Retrospective record review, Incidents, Patient safety, Harvard medical practice study method

## Abstract

**Background:**

In contrast to other safety critical industries, well-developed systems to monitor safety within the healthcare system remain limited. Retrospective record review is one way of identifying adverse events in healthcare. In proactive patient safety work, retrospective record review could be used to identify, analyze and gain information and knowledge about no-harm incidents and deficiencies in healthcare processes. The aim of the study was to evaluate retrospective record review for the detection and characterization of no-harm incidents, and compare findings with conventional incident-reporting systems.

**Methods:**

A two-stage structured retrospective record review of no-harm incidents was performed on a random sample of 350 admissions at a Swedish orthopedic department. Results were compared with those found in one local, and four national incident-reporting systems.

**Results:**

We identified 118 no-harm incidents in 91 (26.0%) of the 350 records by retrospective record review. Ninety-four (79.7%) no-harm incidents were classified as preventable. The five incident-reporting systems identified 16 no-harm incidents, of which ten were also found by retrospective record review. The most common no-harm incidents were related to drug therapy (n = 66), of which 87.9% were regarded as preventable.

**Conclusions:**

No-harm incidents are common and often preventable. Retrospective record review seems to be a valuable tool for identifying and characterizing no-harm incidents. Both harm and no-harm incidents can be identified in parallel during the same record review. By adding a retrospective record review of randomly selected records to conventional incident-reporting, health care providers can gain a clearer and broader picture of commonly occurring, no-harm incidents in order to improve patient safety.

## Background

Safety critical industries such as the aviation and nuclear power industries have gone far beyond healthcare services in their efforts to develop systems to monitor and improve safety. It has become widely accepted in these industries that every major incident is preceded by a number of smaller incidents, and that studying these can reveal system weaknesses that can be improved, thus reducing the risk of incidents or property loss. These practices are derived from early theories of accident causation developed by H.W. Heinrich in the nineteen thirties [1]. Heinrich´s models for accident causation have been further developed, for example as in the ILCI Loss Causation Model [2]. This model indicates that losses begin with lack of control. With lack of control, underlying causes such as lack of training or inadequate tools and equipment are allowed to exist. These underlying causes lead to immediate causes, which are substandard conditions, or the performance of substandard practices. Immediate causes lead to the incident itself, and conclude with the loss. The loss may be to people, property, product, the environment, or the organization’s ability to provide its services. The ILCI model emphasizes the importance of managers to evaluate the management systems that influence human behavior rather than to blame individuals for committing substandard acts or allowing substandard conditions to exist [2].

Heinrich also developed a pyramid shaped model to explain the relationship of near-miss and accidents to minor and major incidents. It was Heinrich’s belief that minor incidents must be prevented in order to eliminate the possibility of reaching each successive level of the pyramid. By studying and categorizing near misses, such substandard conditions can be detected and improved. Systems built on these principles for reporting and categorization of near misses has been developed for other safety critical industries [3]. These safety critical industries combine information from incidents in decades of analyses that have resulted in knowledge of the importance of safe work practices and safety barriers [4-6].

In contrast, similar well-developed systems to monitor patient safety within the healthcare system remain limited [5]. Despite the past decade’s focus on patient safety work, Baines et al. [7] found that the risk of experiencing a preventable adverse event (AE) was higher in 2008 than in 2004, showing patient safety as a persistent problem.

Although there are different methods of identifying incidents in healthcare, experience shows that the most commonly used method, clinical incident-reporting, only identifies a small percentage of incidents, thus limiting opportunities to learn of the nature and contributing factors of incidents [8-10]. Reasons for not reporting incidents are, for example, lack of time, interruption in workflow, fear and lack of trust [11-16]. Retrospective record review (RRR) is one way of identifying deficiencies in healthcare processes. It is a frequently used method in healthcare practice to identify AEs [5,17,18], and in this respect superior to different incident-reporting systems [8,9,19-21].

The World Health Organization has defined an incident as an event or circumstance that could have or has resulted in unnecessary harm to a patient. An AE is an incident resulting in harm to a patient, whereas a no-harm incident is defined as an event that reaches the patient but results in no discernible harm [22].

We hypothesized that RRR, beside its documented advantage in the detection of AEs, could also be a valuable tool to identify and characterize no-harm incidents. Our aim was to evaluate RRR for the detection of no-harm incidents and compare findings with five conventional incident-reporting systems.

## Methods

### Study design

A random sample of 350 admissions was selected from 3701 inpatient admissions during 2009 at a 52-bed Orthopedic Department at a university hospital in Stockholm, Sweden. Both elective and acute admissions were included in the study. We reviewed the healthcare process during the care of the patient. The study emanates from a previous study that compared the ability of two RRR methods, the Harvard Medical Practice Study (HMPS) method and the Global Trigger Tool (GTT) to identify AEs in the same sample of admissions [23]. In the present study the HMPS method was evaluated for the detection of no-harm incidents.

The inclusion criteria were that no-harm incidents had to be related to care provided by the Orthopedic Department, and the meeting of any one of the following criteria:

(i) The no-harm incident was to have been caused within 30 days before index admission, and detected during index admission.

(ii) The no-harm incident had to be caused and detected during index admission.

(iii) The no-harm incident was to have been caused during index admission and detected within 30 days of index discharge from the Orthopedic Department at an inpatient or outpatient visit.

The hospital’s computerized medical record system – referred to as a “record” in this article - was the source of all data since it included documentation from all healthcare professionals. In addition to reviewing index admissions and readmissions, a review of outpatient notes for no-harm incidents connected to the inpatient index admission was carried out.

### Review process

The review team consisted of one registered nurse (RN) and two physicians. The RN (MU) was highly experienced in using screening criteria, and well informed of the orthopedic context. One physician was a senior orthopedic surgeon (OM) experienced in reviewing records. The other physician had no specific orthopedic competence or RRR experience, but had expertise in patient safety (KPH).

To ensure validity, the review process was standardized in a written manual. Detailed examples were developed, discussed and defined by the team prior to the start of the study. In the familiarizing process, each team member independently reviewed 11 written training records followed by a consensus process allowing all involved to engage in discussions of no-harm incident assessment.

RRR based on the method used in the original HMPS [24,25], is a two-stage review process to identify the incidence of AEs using 18 screening criteria. Over the years, modifications of the original method have been made in subsequent studies; e.g. by adding preventability to the review process [26-29].

A two-stage RRR was performed according to HMPS methodology [25,27,29]. In review Stage 1, all records from the random sample were reviewed by the orthopedic nurse. She screened the index admission plus/minus 30 days for the presence of one or more of 18 predefined screening criteria. For every screening criterion detected, a judgment was made by the RN on whether it reflected the presence of a potential no-harm incident or not. This was performed parallel to screening for AEs [23]. The finding was briefly described in a study database to facilitate the next step of the review process. Only records with potential no-harm incidents were forwarded to the physicians for review. Each physician reviewed half the records forwarded by the RN. In addition, every tenth record was reviewed double-blinded by both physicians, which consequently demonstrated inter-rater reliability.

In review Stage 2, the two physicians each performed an independent review. Initially, an assessment of healthcare causation was performed using a 6-point scale [24,28]. Healthcare causation implies the actions of individual healthcare workers as well as the systems and care processes used in delivering healthcare rather than the patient’s underlying illness. It includes both acts of omission (for example, failure to diagnose or treat) and acts of commission (for example, incorrect diagnosis or treatment, or poor performance) [26]. Only potential no-harm incidents with a score of four or higher (i.e. greater than 50% likelihood of healthcare causation) were classified as no-harm-incidents. The physicians then judged if the no-harm incident was considered preventable or not. A preventable no-harm incident was defined as an event in healthcare management due to failure to follow accepted practice at the level of the individual or of the system [28]. A similar 6-point scale was used and a score of four or higher (i.e. more than 50% likelihood) meant that the no-harm incident was considered preventable [28].

The physicians documented the related screening criteria for each no-harm incident. If a record contained more than one no-harm incident, each was reviewed separately, and the associated screening criterion documented separately. In accordance with the HMPS methodology, supplementing assessments were performed for an overview of no-harm incidents. First, no-harm incidents were classified into eight categories describing their nature (e.g. drug related). Also, place of occurrence (e.g. Emergency Department) and contributing factors [30] (e.g. team factors) were documented. The contributing factors that influenced practice were classified according to the framework by Vincent et al. [30] and were adapted to the no-harm incidents.

In order to check that no potential no-harm incident had been excluded by the RN in review Stage 1, every tenth admission that had not been forwarded to a physician for review was screened by one of the two physicians (OM). If the physician found any additional no-harm incidents, they were added. Similarly, any no-harm incident found by the physicians during review Stage 2 was added. These two steps constituted the validation of the RN review.

A comparison between RRR and one local and four nationwide systems for incident-reporting regarding the 350 admissions was also carried out. Documentation of no-harm incidents from the five incident-reporting systems was identified by two RNs (KS and MU). In Sweden each person has a unique personal code number. The five systems are not confidential to the involved healthcare clinic, enabling the investigation of no-harm incidents connected to the same patient sample. The five systems were: 1) the obligatory clinical incident-reporting system at the hospital, 2) hospital reports, named Lex Maria, according to Swedish legislation, 3) malpractice claims reported to the Swedish County Council’s Mutual Insurance Company, 4) the Medical Responsibility Board and 5) patient/relative complaints reported to the Patient Advisory Committee (obligatory in every county council according to Swedish legislation).

Ethical approval was obtained from the Regional Ethics Committee of Stockholm (Number 2008/951-31/3).

### Statistical analysis

Categorical data are presented as frequency counts and percent. Continuous data are presented as median and range. The Mann-Whitney U-test and the Chi-squared test, when appropriate, were used to compare data between groups. The level of significance was set at 0.05. Analysis was carried out using StatView® v5.0.1 (SAS Institute Inc., SAS Campus Drive, Cary, NC, USA) and Excel 2007.

## Results

No records were excluded from review due to missing documentation.

In review Stage 1, the RN found 296 incidents in 156 admissions. These included both no-harm incidents and AEs.

The physicians found no no-harm incidents to add to those already found by the RN in the two validation stages of the RN review process. The physicians’ assessments of the 11 no-harm incidents reviewed by both were coherent in all of the cases concerning healthcare causation and preventability.

After review stage 2, 118 no-harm incidents were identified in 91 (26.0%) of the 350 admissions (range 0-3 no-harm incident per admission). The demographics for patients with and without no-harm incidents are displayed in Table 1.

**Table 1:** Demographic variables of the study sample of 350 admissions

**Characteristics**	**Admissions with no-harm incidents**	**Admissions without no-harm incidents**	**p**
Admission, number (%)	91 (26.0)	259 (74.0)	
Women, number (%)	57 (62.6)	144 (55.6)	0.73
Age (years), median (range)	72 (17-96)	67 (15-97	0.08
Acute admissions, number (%)	66 (72.5)	184 (71.0)	0.48
Length of stay (days), median (range)	6 (1-55)	4 (1-21)	<0.0001

Of the identified 118 no-harm incidents, 79.7% (n = 94) were classified as preventable. Of these 96.6% (n = 114) were identified during inpatient care. Four (3.4%) were discovered during outpatient visits in connection with the randomized index admission.

Sixteen no-harm incident reports for 15 patients were found in the five clinical incident-reporting systems. Fifteen no-harm incidents were found via the local clinical incident-reporting system and nine of these had also been identified by RRR. One no-harm incident was found in the incident-reporting systems outside the Department´s own reporting system. This single no-harm incident was also identified by RRR. Nine of the 16 no-harm incidents found in the five incident-reporting systems were related to drugs.

### Positive predictive value of screening criteria

After review Stage 2, a total of 462 (range 1-11 per record) screening criteria were identified in 195 records (Table 2). The Positive Predictive Value (PPV) of screening criteria was defined as the number of times a specific screening criterion identified a no-harm incident divided by the total number of times the screening criterion was found. The PPV for no-harm incidents was 27.7% (range 0.0-82.8). The screening criterion with the highest PPV for no-harm incidents was “any other undesirable outcome not covered above”.

**Table 2:** **Screening criteria modified after the Harvard medical practice study**[27]**ordered by positive predictive value for no-harm incident**

**Screening criteria**	**Positive screening criteria n (%)**	**Screening criteria related to no-harm incident n**	**PPV for no-harm incident%**
Any other undesirable outcome not covered above	87 (18.8)	72	82.8
Adverse drug reaction	33 (7.1)	15	45.5
Hospital-incurred patient injury or no-harm incident	129 (27.9)	32	24.8
Dissatisfaction with care documented in the patient’s medical record	16 (3.4)	3	18.8
Other patient complication	13 (2.8)	2	15.4
Unplanned return to the operating room	10 (2.1)	1	10.0
The index admission was an unplanned admission related to previous healthcare management within 30 days	25 (5.4)	1	4.0
Unplanned readmission after discharge from index admission within 30 days including outpatient visits	85 (18.3)	3	3.5
Unplanned transfer from general care to intensive care	5 (1.0)	-	-
Unplanned removal, injury or repair of an organ during surgery	5 (1.0)	-	-
Development of neurological deficit not present on admission	5 (1.0)	-	-
Healthcare associated infection or sepsis	38 (8.2)	-	-
Cardiac or respiratory arrest	2 (0.4)	-	-
Documentation or correspondence indicating litigation	3 (0.6)	-	-
Inappropriate discharge to home	5 (1.0)	-	-
Unplanned transfer to another acute care hospital	1 (0.2)	-	-
Unexpected death	0 (0.0)	-	-
Injury related to abortion or delivery	NA		
Total	462 (100)	129	27.9

*NA*, Not applicable.

*PPV*, Positive predictive value.

### Nature and preventability of no-harm incidents

The nature and preventability of the no-harm incidents are shown in Table 3. Drug-related no-harm incidents related to nursing care or medical care were found in 66 of the cases and 58 (87.9%) were considered preventable. Examples of no-harm incidents in the category “drug related to nursing care” are events when important medication was prescribed but not administered, and in the category “drug related to medical care” when a prescription of an important medicine was not made in accordance to standard care. Examples of important medication are antibiotic and thrombosis prophylaxis. In the category “nursing care excluding drugs”, patient falls were the most common. Missed hygiene precaution in patients with Methicillin-resistant Staphylococcus aureus and missed execution of a planned X-ray are examples of system related, no-harm incidents.

**Table 3:** Nature of no-harm incidents

**Nature of no-harm incidents**	**Total n (%)**	**Preventable n (%)**
Drug, related to nursing care	40 (33.9)	40 (100)
Drug, related to medical care	26 (22.0)	18 (69.2)
Nursing care, excluding drugs	18 (15.3)	13 (72.2)
System related	12 (10.2)	12 (100)
Anaesthesia related	10 (8.5)	3 (30.0)
Surgical and invasive procedures	7 (5.9)	4 (57.1)
Diagnostics related	3 (2.5)	3 (100)
Treatment excluding drugs and surgical procedures	2 (1.7)	1 (50.0)
Total	118	94

### Place of occurrence of no-harm incidents

Eighty two (69.5%) no-harm incidents occurred on the wards. Twenty eight (23.7%) were related to anesthesia and surgery in the perioperative phase of care. The Emergency Department was involved in five (4.2%) no-harm incidents, the Recovery Unit in two (1.7%) and one (0.8%) no-harm incident occurred at the Orthopedic Outpatient Unit.

### Contributing factors for no-harm incidents

The contributing factors for no-harm incidents are listed in Table 4. Team factors were the most common contributing factor contributing to 100 (41.2%) of the no-harm incidents. Team factors include verbal and written communication, supervision and seeking help, and team leadership [30].

**Table 4:** **Contributing factors**^**a **^**influencing no-harm incidents**

**Main category**	**n**
Team factors, e.g. verbal and written communication	100
Task factors, e.g. availability and use of protocols, availability and use of test results	97
Work environmental factors, e.g. design, availability and maintenance of equipment, staffing levels and skills mix	23
Other/unknown	12
Individual (staff) factors, e.g. knowledge and skills, physical and mental health	7
Organizational and management factors, e.g. organizational structure, policy, standards and goals	4
Institutional context factors, e.g. economic and regulatory context	0
Total	243^b^

^a^ Modified from Vincent, Taylor-Adams & Stanhope, 1998 [30].

^b^ The number of contributing factors is higher than the number of no-harm incidents because the reviewers were allowed to choose more than one contributing factor for each no-harm incident.

## Discussion

We found that no-harm incidents occurred in as many as a quarter of orthopedic admissions and were preventable to a high degree (79.7%). The no-harm incidents were identified mainly within three areas: drug-related; patient falls without harm; and system-related; in many cases lack of compliance to evidence-based guidelines i.e. missed hygienic precaution. Considerably more no-harm incidents were identified by using RRR compared to five local and nationwide incident-reporting systems. Our study confirms a small overlap between different methods for identifying incidents [31-33].

### Collection of safety information and the use of RRR

We found that RRR could be a valuable tool to identify safety information about no-harm incidents when used in a structured way through random samples and implicit review. RRR identified more no-harm incidents compared to other incident-reporting systems. This is in line with other RRR studies that have been found to identify more AEs compared with other safety information methods [9,10,17,21,34].

Collecting data is the first step of an organisational learning process; and data collection is necessary to assess risks and incident rates, and to provide the basis to prioritise where to deploy resources and how to implement change, as well as to later monitor progress in patient safety outcomes [35,36]. To measure patient safety and to follow up on safety initiatives is difficult. Much of our learning in healthcare has come from individual clinical cases and not from aggregated data, the latter which may be needed to prioritise and evaluate safety efforts [35]. A problem when performing systematic safety analysis is that when different methods are used to collect patient safety information, the outcomes are not easily comparable. Often information is collected at different levels in the healthcare system and by different organizations. A systematic comparison is difficult since different methods yield different information about incidents [15]. Furthermore, some of the information is not easily accessible to healthcare leaders and clinicians, and the identification and dissemination of information is often delayed, which complicates matters.

Other safety critical industries have a more evolved approach to the analysis and study of incident data, having realized that incident-reporting is complementary to other measurements and only one part of a wide range of safety assessments, measurements and monitoring [37]. Several methods to identify and follow-up safety issues are needed and complementary. When combined with other patient safety reporting systems, RRR may provide a clearer and broader picture of the no-harm incidents occurring in healthcare, to support study and point out safety problems that need to be prioritized for implementing safety interventions [9].

RRR does not need to be resource intensive. In the present study the median review time for the RN to identify no-harm incidents was three minutes per record. Results from RRR can be further analyzed within the organization in the same way as information from clinical incident-reporting systems, and can form the basis of a root cause analysis to deepen the understanding of incidents. If no-harm incidents are included when using RRR to detect AEs, the validity of no-harm incident data, learning, patient safety outcomes, and proactive work may increase. The strength is that implicit and explicit record reviews used retrospectively or prospectively, are commonly used methods in health care for safety and quality work, by advantageously using currently available data [38]. The method can be repeated over time and specific incident types can be targeted, and not just the overall incident rate [15].

Another RRR method, the GTT, may also be useful for identifying and categorizing no-harm incidents [39,40]. The GTT, used in several hospitals in many countries in clinical patient safety work uses the National Coordinating Council for Medication Error Reporting and Prevention (NCC MERP) scale of severity, but excludes the levels that comprise risks and no-harm incidents. Since RRR seems useful even for identifying no-harm incidents, we believe that with minimal effort GTT review teams can also include no-harm incidents while reviewing records for AEs; and by adding appropriate parts of the NCC MERP scale the no-harm incidents can be categorized according to severity [41].

### The importance of no-harm incidents

An important reason to analyse and act on common and often preventable no-harm incidents aside from reducing the number of no harm incidents, is that an incident not leading to an AE in one case might be a sentinel of serious system defects that could result in major harm in the next case, e.g. pulmonary embolism or wound infection due to lack of prophylactic treatment. The same contributing factors are present in both cases but the outcomes may vary. No-harm incidents are a valuable source of information in healthcare, since they provide a source of low cost learning about system failures. A system of organizational learning that relies on the identification of failures before, rather than after, an incident occurs is preferred [42]. Other safety critical industries have evolved considerably over time to focus on both proactive and reactive indicators [37]. Our findings of no-harm incidents point to underlying factors that put patients at risk, and are important signals to the health care staff as well as to the hospital board that routines are insufficient. It is important to continue giving the problem attention, and safety interventions are needed to reduce both the high frequency of no-harm incidents as well as AEs. The fact that an incident does not cause an AE, but remains a no-harm incident, may depend on various barriers and rescue strategies by the staff, both in terms of counter measures and increased monitoring of patients, thus affecting the workload of the staff while preventing patients from AEs [43].

The comparison between healthcare and other safety critical industries must be made with caution. Organizations such as the aviation and nuclear power industries have been pressured by political, social and humanitarian movements to improve and maintain safety. Work in such dangerous environments has made staff members and management aware of the need to discuss, analyze, manage, and provide adequate resources for safety work. In healthcare severe outcomes in relation to care is not always as obvious as in e.g. an airplane crash, and the incidents are more spread out in time and location. Success factors in most safety critical industries have been based on thorough analyses of context and culture-specific factors contributing to incidents. They have led to strategies such as paying great attention and providing resources to prepare and develop employee skills as a team, standardizing requirements in equipment, education and practice. These are all matters that have not attracted much attention within the healthcare system until the beginning of the past decade [15].

### Contributing factors

A common contributing factor was the defiencies of communication of vital information within and between teams caring for the patients. This finding points to the importance of team training and the improvement of information transfer and documentation. Unclarity regarding task, including e.g. task design and structure, and the availability and use of protocols were other common contributing factors. Difficulties in implementing and following protocols are known problems in the healthcare system [44-46]. Table 4 gives an indication of the variety and complexity of contributing factors.

### The use of the screening criteria to identify no-harm incidents

Some of the screening criteria were never detected, as they per definition describe AEs, e.g. healthcare associated infections. Most no-harm incidents were ascribed to the unspecific screening criterion “any other undesirable outcome not covered above”, which in our view is a weakness of the HMPS method when used for no-harm incidents. We suggest that the screening criteria should be revised in order to strengthen the method for identifying no-harm incidents.

### Limitations

This study has limitations. No-harm incidents were identified at a single orthopedic department, which affects generalizing our findings to other areas of healthcare. However, the data collection methods, the types of data found and analyzed are likely to be available at most hospitals. Nor can we say that we have found the “true values”, since such do not exist. Our results are based on review by only one RN, and the inter-rater agreement data between secondary reviewers are limited. We do not believe that our main conclusions that no-harm incidents are common and often preventable are affected by this limitation. RRR in itself also has limitations. Poor documentation or a lack of documentation, the major obstacle in a retrospective review, could lead to underestimation thus causing incidents to go unnoticed. Untangling incidents from the patient’s underlying disease processes can be difficult, and the reviewers’ experience, safety attitudes and clinical experience could lead to differences in judgments between reviewers [47]. The retrospective design and record notes as the only information source limits a complete analysis of contributing factors. In order to gain deeper knowledge, prospective record review or root cause analyses are preferred.

## Conclusions

No-harm incidents are common and often preventable. Retrospective review of patient records may be a useful tool for identifying no-harm incidents, and a valuable complement to other safety information sources in detecting system failures that affect patient safety.

## Abbreviations

AE: Adverse event; HMPS: Harvard Medical Practice Study; GTT: Global Trigger Tool; NA: Not applicable; NCC MERP: National Coordinating Council for Medication Error Reporting and Prevention; PPV: Positive predictive value; RN: Registered nurse; RRR: Retrospective record review.

## Competing interests

None of the authors have competing interests. Financial support was provided through the Swedish Vinnvård programme for MU and KPH and by FORSS – the research council of Southeast Sweden and the County Council of Kalmar for KS. Vinnvård and FORSS have neither been involved in any part of the study, nor in writing the manuscript, or in the decision to submit the manuscript for publication.

## Authors’ contributions

KS: Main author, responsible for study design, analyses, manuscript drafting and intellectual content. MU: Project supervisor, responsible for study design, acquisition of data, analysis, manuscript drafting and intellectual content. OM: Responsible for study design, acquisition of data, and intellectual content. JP: Manuscript drafting and intellectual content. KPH: Responsible for study design, acquisition of data, manuscript drafting, and intellectual content. LN: Responsible for study design, manuscript drafting and intellectual content. All authors read and approved the final manuscript.

## Pre-publication history

The pre-publication history for this paper can be accessed here:

http://www.biomedcentral.com/1472-6963/13/282/prepub
